# Demonstration of translation elongation factor 3 activity from a non-fungal species, *Phytophthora infestans*

**DOI:** 10.1371/journal.pone.0190524

**Published:** 2018-01-04

**Authors:** Maria K. Mateyak, Justyna K. Pupek, Alexandra E. Garino, McCllelan C. Knapp, Sarah F. Colmer, Terri Goss Kinzy, Stephen Dunaway

**Affiliations:** 1 Department of Biochemistry and Molecular Biology, Robert Wood Johnson Medical School, Rutgers, the State University of New Jersey, Piscataway, NJ, United States of America; 2 Department of Biology, Drew University, Madison, NJ, United States of America; University of Lethbridge, CANADA

## Abstract

In most eukaryotic organisms, translation elongation requires two highly conserved elongation factors eEF1A and eEF2. Fungal systems are unique in requiring a third factor, the eukaryotic Elongation Factor 3 (eEF3). For decades, eEF3, a ribosome-dependent ATPase, was considered “fungal-specific”, however, recent bioinformatics analysis indicates it may be more widely distributed among other unicellular eukaryotes. In order to determine whether divergent eEF3-like proteins from other eukaryotic organisms can provide the essential functions of eEF3 in budding yeast, the eEF3-like proteins from *Schizosaccharomyes pombe* and an oomycete, *Phytophthora infestans*, were cloned and expressed in *Saccharomyces cerevisiae*. Plasmid shuffling experiments showed that both *S*. *pombe* and *P*. *infestans* eEF3 can support the growth of *S*. *cerevisiae* in the absence of endogenous budding yeast eEF3. Consistent with its ability to provide the essential functions of eEF3, *P*. *infestans* eEF3 possessed ribosome-dependent ATPase activity. Yeast cells expressing *P*. *infestans* eEF3 displayed reduced protein synthesis due to defects in translation elongation/termination. Identification of eEF3 in divergent species will advance understanding of its function and the ribosome specific determinants that lead to its requirement as well as contribute to the identification of functional domains of eEF3 for potential drug discovery.

## Introduction

Translation is a highly conserved process during which proteins are synthesized from messenger RNA. This process is divided into the four phases of initiation, elongation, termination and ribosome recycling, each of which requires a specialized set of soluble protein factors (reviewed in [1]). During the initiation phase of translation, eukaryotic initiation factors facilitate the binding of an 80S ribosome at the start codon of the mRNA. This is followed by a repetitive cycle of aminoacyl-tRNA (aa-tRNA) delivery, peptide bond formation and ribosomal translocation during the elongation phase. In most eukaryotic organisms, translation elongation is catalyzed by two soluble elongation factors (eEFs), eEF1A and eEF2. eEF1A, the functional homolog of bacterial EF-Tu, is a G-protein that binds and recruits aa-tRNAs to the A-site of the ribosome. When a codon-anticodon match occurs, the ribosome stimulates eEF1A-mediated GTP hydrolysis resulting in the release of inactive GDP-bound eEF1A from the ribosome and accommodation of the aa-tRNA into the A-site. Following peptide bond formation, eEF2, the homolog of the bacterial GTPase EF-G, catalyzes the translocation of the peptidyl-tRNA from the A-site to the P-site of the ribosome thereby positioning the next codon for decoding. When a stop codon is encountered by the ribosome, release factors release the polypeptide from the ribosome and the ribosomal subunits are recycled for another round of protein synthesis.

Fungal translation elongation has long been considered unique among eukaryotes in its requirement for a third elongation factor, eEF3. eEF3 is a ribosome-dependent ATPase required for *in vitro* translation elongation assays when using ribosomes purified from yeast [2]. In contrast, *Saccharomyces cerevisiae* eEF1A and eEF2 alone catalyze translation elongation with rat liver ribosomes [3]. Therefore, it is likely a ribosome-specific determinant that establishes the need for eEF3. The reason why ribosomes from yeast require eEF3 and the function of eEF3 itself are not well understood. In one study, eEF3 facilitated the release of de-acetylated tRNA from the E-site of the ribosome [4]. eEF3 also stimulates eEF1A-mediated binding of cognate aa-tRNA to the A-site [5, 6] potentially through a direct interaction with eEF1A. [7, 8]. *in vivo* experiments confirm the important role of eEF3 in yeast protein synthesis. eEF3 is encoded by an essential gene in *S*. *cerevisiae* and strains harboring either temperature sensitive or mutant forms of eEF3 display protein synthesis and translation elongation defects [7–10]. eEF3 orthologues from other yeasts including *Candida albicans*, another ascomycete and *Cryptococcus neoformans*, a basidiomycete, can complement the loss of eEF3 in *S*. *cerevisiae* suggesting that the function of eEF3 is likely conserved among fungi [11, 12].

Structural studies of eEF3 identified five domains: an amino-terminal HEAT repeat followed by a four-helix bundle domain and two ATP-binding cassette (ABC) domains, the second of which is interrupted by a chromodomain insertion [13]. A cryo-EM reconstruction of the eEF3-ATP-post-translocation 80S ribosome complex demonstrated that eEF3 binds the ribosome near the E-site in agreement with its proposed function in E-site tRNA release [13]. The chromodomain insertion is proposed to interact with the ribosome and stabilize the ribosomal L1 stalk in an open conformation which may facilitate tRNA release. Mutations in this domain, however, show significant reduction in ATPase activity without affecting overall ribosome binding [10].

Recent bioinformatic analysis identified potential eEF3 orthologues in multiple non-fungal, lower eukaryotic species [14, 15]. These eEF3-like protein sequences are as similar to *S*. *cerevisiae* eEF3 as the functionally complementary *C*. *neoformans* eEF3, suggesting that these putative eEF3s are likely to maintain at least a subset of eEF3 functions. In this study, we present the first direct evidence suggesting that functional eEF3 orthologues exist outside the fungal kingdom. We have expressed the eEF3 orthologue from *Schizosaccharomyes pombe* and the oomycete *Phytopthora infestans*, the causative agent of late potato blight, in *S*. *cerevisiae* and showed that either can provide the essential functions of eEF3. *in vitro* studies demonstrated that *P*. *infestans* eEF3, like *S*. *cerevisiae* eEF3, possesses ribosome-stimulated ATPase activity.

## Materials and methods

### Plasmid construction

*S*. *cerevisiae* eEF3: The sequence encoding an N-terminal 6x-His tagged *S*. *cerevisiae* eEF3 was cloned as a *Bam*HI fragment from plasmid pTKB1142 into plasmid pTKB328 (*CEN LEU2*) where it is expressed from the *S*. *cerevisiae TEF5* promoter (pTKB1263).

*Schizzosaccharomyces pombe* eEF3: The *S*. *pombe tef3*^*+*^ gene was amplified by PCR from fission yeast genomic DNA prepared using the Epicentre Masterpure Yeast DNA Purification Kit. The PCR was performed using the following primers: Forward Primer-5’ ATTGTTTGATCAATGCATCATCATCATCATCATTCTGCAAAGAGTGA-GAATAA-3’, Reverse Primer-5’CTATTAGGGCCCTTACAGGTCACTGACCTC-3’. The PCR product encoding N-terminal 6x-His tagged *S*. *pombe* eEF3 was cloned into the *Bam*HI and *Apa*I restriction sites of the pTKB328 vector and is expressed from the *S*. *cerevisiae TEF5* promoter (TKB1269).

*P*. *infestans* eEF3: The sequence encoding *P*. *infestans* eEF3 (NCBI Reference Sequence XP_002906761.1) was synthesized as two codon-optimized gblock gene fragments with an N-terminal 6x-His tag (Integrated DNA Technologies). These two gene fragments were cloned into plasmid pTKB328 using Gibson assembly (New England Biolabs) such that N-terminal 6x-His tagged *P*. *infestans* eEF3 is expressed from the *S*. *cerevisiae TEF5* promoter (pTKB1264). The *Sac*1-*Xho*I fragment of pTKB1264 containing the *TEF5* promoter and *P*. *infestans* eEF3 was cloned into pTKB372 (2μ *LEU2*) creating pTKB1268

### Strains and growth conditions

*S*. *cerevisiae* strains used in this study are listed in Table 1. Yeast cells were grown in either yeast extract-peptone-dextrose (YEPD; 1% Bacto-yeast extract, 2% Bacto-tryptone, 2% dextrose) or defined synthetic complete medium (C) lacking the indicated amino acid and supplemented with 2% dextrose as a carbon source. Growth curves were generated by diluting exponentially growing cultures to an A_600_ of 0.1 and then measuring the A_600_ at the indicated time points.

**Table 1:** *S*. *cerevisiae* strains used in this study.

Strain	Genotype	Reference
***TKY1617***	*MATα leu2-3*, *112 trp1-1 can1-100 ura3-1 ade2-1 his3-11*,*15 yef3*:*HIS3 p S*.*cerevisiae YEF3 URA3 CEN*	*[10]*
***TKY1653***	*MAT*α*leu2-3*,*112 trp1-1 can1-100 ura3-1 ade2-1 his3-11*,*15 yef3*::*HIS3 p 6x-His YEF3 TRP1 CEN*	*[10]*
***TKY1786***	*MATα leu2-3*, *112 trp1-1 can1-100 ura3-1 ade2-1 his3-11*,*15 yef3*:*HIS3 pTEF5 6x-His S*. *pombe TEF3 LEU2 CEN*	*This work*
***TKY1787***	*MATα leu2-3*, *112 trp1-1 can1-100 ura3-1 ade2-1 his3-11*,*15 yef3*:*HIS3 pTEF5 6x-His P*. *infestans EF3 LEU2 CEN*	*This work*
***TKY1791***	*MATα leu2-3*, *112 trp1-1 can1-100 ura3-1 ade2-1 his3-11*,*15 yef3*:*HIS3 pTEF5 6xHis S*. *cerevisiae YEF3 LEU2 CEN*	*This work*
***TKY1792***	*MATα leu2-3*, *112 trp1-1 can1-100 ura3-1 ade2-1 his3-11*,*15 yef3*:*HIS3 pTEF5 6x-His P*. *infestans EF3 LEU2 2μ*	*This work*

Heterologous eEF3 proteins were expressed as the only form of eEF3 by plasmid shuffling in TKY1617. Briefly, TKY1617 was individually transformed with the plasmids described above using a lithium acetate method. Transformants were counterselected on media containing 5-fluoroorotic acid to select cells that have lost the complementing *S*. *cerevisiae* eEF3 expressing plasmid.

### Immunoblotting

Yeast cells in mid-log phase growth were lysed with glass beads in 100 mM Tris-HCl pH 8.0, 20% glycerol, 1 mM dithiothreitol and 1 mM phenylmethylsulfonyl fluoride and cell debris was removed by centrifugation. Protein concentration in cell lysates was determined using the BioRad reagent according to the manufacturer’s instructions. Twenty-five micrograms of total protein were separated on a 7.8% SDS-polyacrylamide gel and transferred to a nitrocellulose membrane. The membrane was stained with Ponceau S and immunoblotted with either a monoclonal anti-His antibody (BD Biosciences) or a monoclonal anti-PGK antibody (Novex). Quantitation of immunoblots was performed using ImageQuant software (GE Healthcare).

### Antibiotic sensitivity assays

Yeast strains in exponential growth were diluted to an A_600_ of 0.6 and 200 μl was spread on YEPD plates. Sterile discs were placed on the plates and 10 μL of antibiotic at the indicated concentration was pipetted onto the disc. Plates were incubated at 30°C for 2d at which time the diameter of the zone of growth inhibition surrounding the discs was measured. Antibiotic concentrations were 1 mM cycloheximide, 25 mM hygromycin and 800 mg/mL paromomycin.

### Translation assays

For *in vivo* [^35^S] methionine incorporation assays, yeast strains were grown in liquid culture in C-Met at 30°C to mid-log phase and assayed as previously described [16]. All time points were analyzed in triplicate. Polyribosome extracts were harvested and run on a 7–47% sucrose density gradient as previously described [17]. The areas under the 80S and polyribosome peaks were determined using Image J (National Institutes of Health).

### Purification of 6x-His-eEF3 proteins

The 6x-His tagged *S*. *cerevisiae* eEF3 was purified from TKY1653 as previously described [10]. The 6x-His tagged *P*. *infestans* eEF3 was purified from TKY1792 on a 1 mL HisTrap Column (GE Healthcare) in buffer A (50 mM potassium phosphate, pH 8.0, 1 M KCl, 0.1% Triton X-100, 10% glycerol, 1 mM DTT, and 0.2 mM PMSF plus Complete Protease Inhibitor (Roche)) with 20 mM imidazole. The protein was eluted using a gradient (0–100%) with buffer B (50 mM potassium phosphate, pH 8.0, 1 M KCl, 0.1% Triton X-100, 10% glycerol, 500 mM imidazole, 1 mM DTT, and 0.2 mM PMSF plus Complete Protease Inhibitor) over 20 column volumes. The protein was dialyzed into buffer C (20 mM HEPES, pH 7.5, 500 mM KCl, 0.1% Triton X-100, 0.1 mM EDTA, 10% glycerol, 1 mM DTT, and 0.2 mM PMSF)

### ATP hydrolysis assay

ATP hydrolysis was analyzed using the PiColorLock Gold Phosphate Detection System (Innova Biosciences) according to the manufacturer’s instructions with the following specifications. The reactions were performed at room temperature for 30 min in 50 mM Tris, pH 7.5, 5 mM MgCl2, and 1 mM ATP with varying concentration of eEF3 in the presence or absence of 25 nM crude *S*. *cerevisiae* ribosomes prepared by the method of [18].

### Phylogenetic analysis

A phylogenetic tree based on eEF3 sequences was generated using phylogeny.fr (http://www.phylogeny.fr) [19, 20]. Accession numbers for the sequences used were as follows: *S*. *cerevisiae* (NP_013350.1), *S*. *pombe* (NP_588285.1), *Candida albicans* (XP_711356.2), *Aspergillus fumigatus* (XP_748943.1), *Blastomyces dermatitidis* (EQL38330.1), *Cryptococcus neoformans* (XP_570261.1), *P*. *infestans* (XP_002906761.1), *Saprolegnia diclina* (XP_008620178.1), *Ectocarpus siliculosus* (CBJ25582.1), *Fistulifera solaris* (GAX16349.1), *Chlamydomonas reinhardtii* (XP_001692287.1), *Volvox carteri* (XP_002951155.1), and *Coccomyxa subellipsoidea* (EIE22864.1). Default setting were used except for the inclusion of bootstrapping.

## Results

### eEF3 activity is not unique to fungi

**Fig 1:**
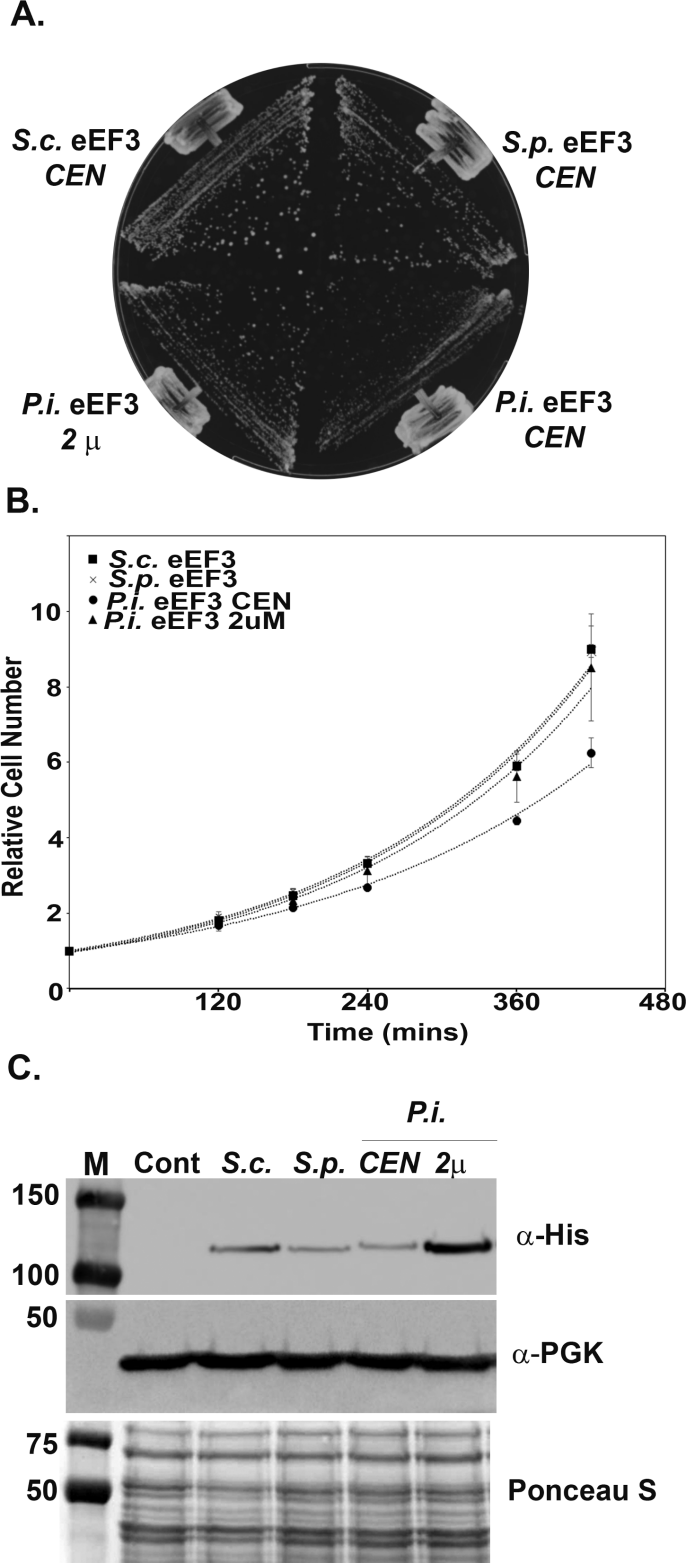
*S*. *pombe (S*.*p*.*)* and *P*. *infestans (P*.*i*.*)* eEF3 complement the loss of *S*. *cerevisiae (S*.*c*.*)* eEF3. (A) Yeast strains expressing eEF3 from the indicated species as the only form of eEF3 were streaked onto YEPD medium and incubated at 30°C for 2 d. CEN–low copy number plasmid/low expression. 2μ –high copy number plasmid/high expression. (B) Growth curves were generated from A_600_ measurements of exponentially growing cultures over the indicated time period. Error bars represent standard error. (C) Whole cell extracts were prepared from the indicated strains, separated by SDS-PAGE, and stained with Ponceau S (bottom panel). The membrane was then cut and immunoblotted with either an anti-His antibody to detect eEF3 or anti-Pgk1 antibody as a loading control. The control lane is an extract from a yeast strain (TKY1617) that does not express epitope tagged eEF3.

While originally designated as a fungal-specific elongation factor, recent bioinformatic analysis identified eEF3-like proteins in several lower eukaryotic species [14, 15]. The most significant regions of conservation between these proteins and *S*. *cerevisiae* eEF3 are found within the two ABC-type ATPase domains. Therefore, it is important to demonstrate that these proteins are not simply related ATPases but that they can provide the essential functions of eEF3. To address this question, the genes encoding eEF3 from an evolutionarily distant ascomycete, *S*. *pombe*, and the putative form of eEF3 from the oomycete, *P*. *infestans*, were cloned into a low copy *S*. *cerevisiae* expression vector with an N-terminal 6x-His tag (materials and methods). *S*. *cerevisiae* eEF3 is approximately 62% identical to *S*. *pombe* eEF3 and 45% identical to *P*. *infestans* eEF3. *S*. *pombe* eEF3 was included in the analysis to broaden the understanding of the evolution of eEF3. Both eEF3 proteins are expressed from the strong *TEF5* promoter and the *P*. *infestans eEF3* gene sequence was codon optimized for expression in budding yeast. The*YEF3* gene encoding *S*. *cerevisiae* eEF3 was also cloned into the same expression vector as a control. The eEF3 expression plasmids were individually transformed into a *S*. *cerevisiae* strain in which the only copy of *YEF3* is untagged and present on a *URA3* plasmid. The ability of *S*. *pombe* or *P*. *infestans* eEF3 to function as the only form of the protein was assessed by growth on 5-FOA containing media which only allows growth of cells that have lost the *URA3* plasmid. Both *S*. *pombe* and *P*. *infestans* eEF3 could support growth of a *yef3Δ* strain (Fig 1A). While expression of *S*. *pombe* eEF3 could support growth at a level similar to *S*. *cerevisiae* eEF3, the strain expressing *P*. *infestans* eEF3 grew at a reduced rate (Fig 1A and 1B). The average doubling time was 137±4 min for the *S*.*c*. eEF3 expressing strain, 135±2 min for the *S*.*p*. eEF3 expressing strain and 162±9 min for the *P*.*i*. eEF3 (CEN) expressing strain. The ability of *P*. *infestans* eEF3 to complement the loss of *YEF3* in *S*. *cerevisiae* suggests that it can perform the essential functions of eEF3 and is a bona fide eEF3 homologue.

The reduced growth rate of cells expressing *P*. *infestans* eEF3 may arise from either reduced protein expression or reduced activity in a heterologous system. Cell lysates were collected and immunoblotted with an anti-His antibody to detect eEF3 expression levels in strains whose growth was supported solely by either *S*. *cerevisiae* eEF3, *S*. *pombe* eEF3 or *P*. *infestans* eEF3 (Fig 1C). The steady state levels of both *S*. *pombe* eEF3 and *P*. *infestans* eEF3 were comparable but lower than that of the native *S*. *cerevisiae* eEF3 protein. These data suggest that there is a threshold level of eEF3 activity required for wild-type growth. To determine if increasing the expression of *P*. *infestans* eEF3 would rescue the slow growth phenotype, 6x-His tagged *P*. *infestans* eEF3 expressed from the same promoter was cloned into a high copy expression plasmid and its expression level and ability to support growth of a *yef3Δ* strain were determined as described above. Over expression of *P*. *infestans* eEF3 (average of 2.2-fold over *S*. *cerevisiae* eEF3) could rescue the growth of *yef3Δ* cells to wild-type (*S*. *cerevisiae* eEF3) levels with an average doubling time of 138±14 min.(Fig 1B and 1C).

### *P*. *infestans* eEF3 shows ribosome-stimulated ATPase activity

**Fig 2:**
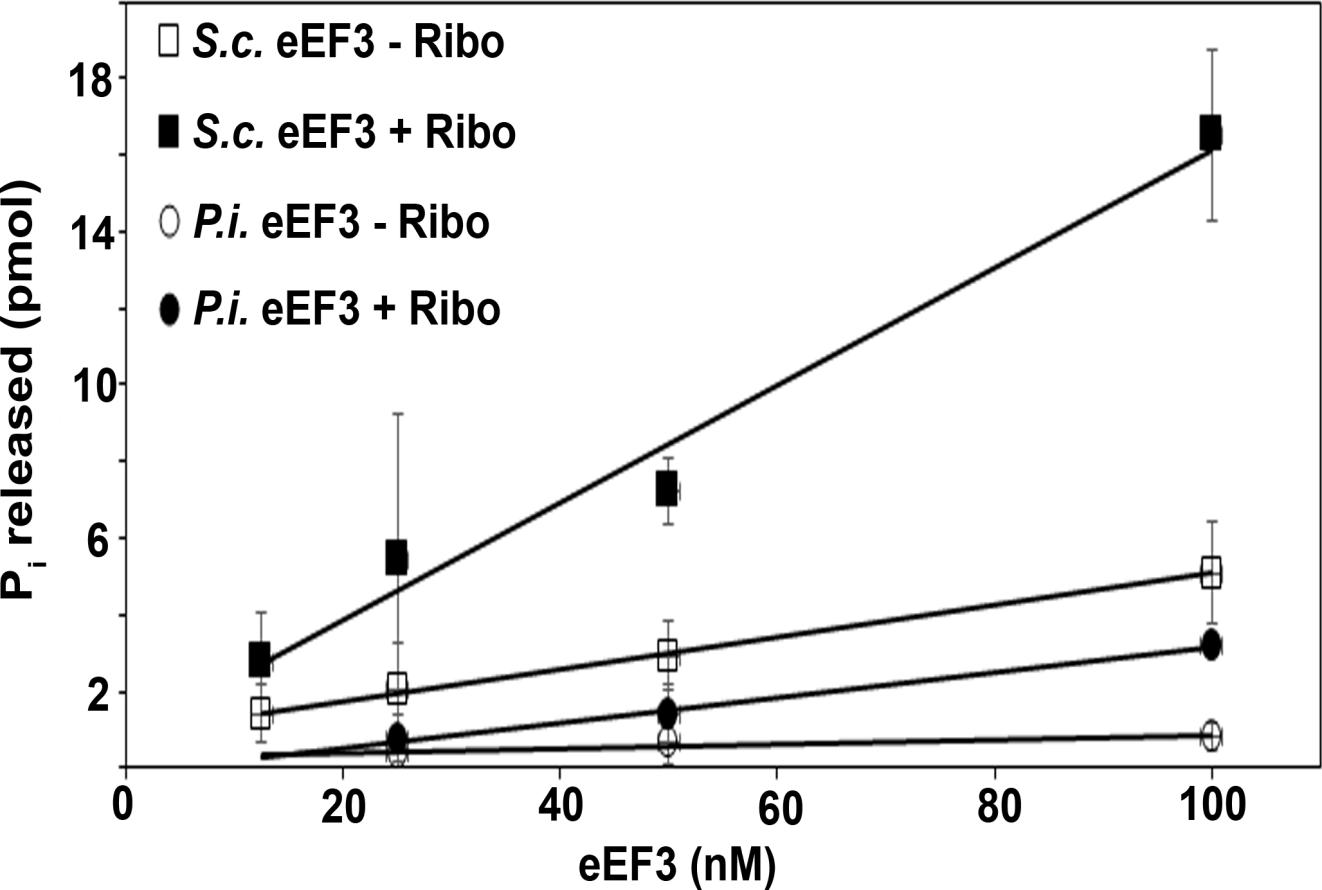
*P*. *infestans* eEF3 possesses ribosome-stimulated ATPase activity. ATPase activity of *S*. *cerevisiae* (■) and *P*. *infestans* (●) eEF3 was assayed in the presence (filled) or absence (empty) of *S*. *cerevisiae* ribosomes. The P_i_ released was measured using the PiColorLock Gold Phosphate Detection System. Reactions included varying amounts of eEF3, 25 nM ribosomes and 1 mM ATP and were carried out at room temperature for 30 min.

A key characteristic of *S*. *cerevisiae* eEF3 is its ribosome-stimulated ATPase activity. To determine if *P*. *infestans* eEF3 demonstrated similar activity, *P*. *infestans* eEF3 was expressed and purified from budding yeast and its activity was compared to *S*. *cerevisiae* eEF3. *S*. *cerevisiae* eEF3 has a low basal ATPase activity that was stimulated 3.2-fold at 100 nM eEF3 by the inclusion of ribosomes as reported previously (Fig 2, [10, 21, 22]). The activity of *P*. *infestans* eEF3 in the absence of ribosomes was very low compared to *S*. *cerevisiae* eEF3 (6.2-fold lower) at the same concentration. The inclusion of *S*. *cerevisiae* ribosomes could stimulate *P*. *infestans* eEF3 activity (3.9-fold) which is similar to the observed ribosome stimulation of *S*. *cerevisiae* eEF3 activity. While the ribosome-stimulated *P*. *infestans* eEF3 activity was concentration dependent, its activity was still 5.2-fold lower than *S*. *cerevisiae* eEF3.

### Cells expressing *P*. *infestans* eEF3 exhibit translation defects

**Fig 3:**
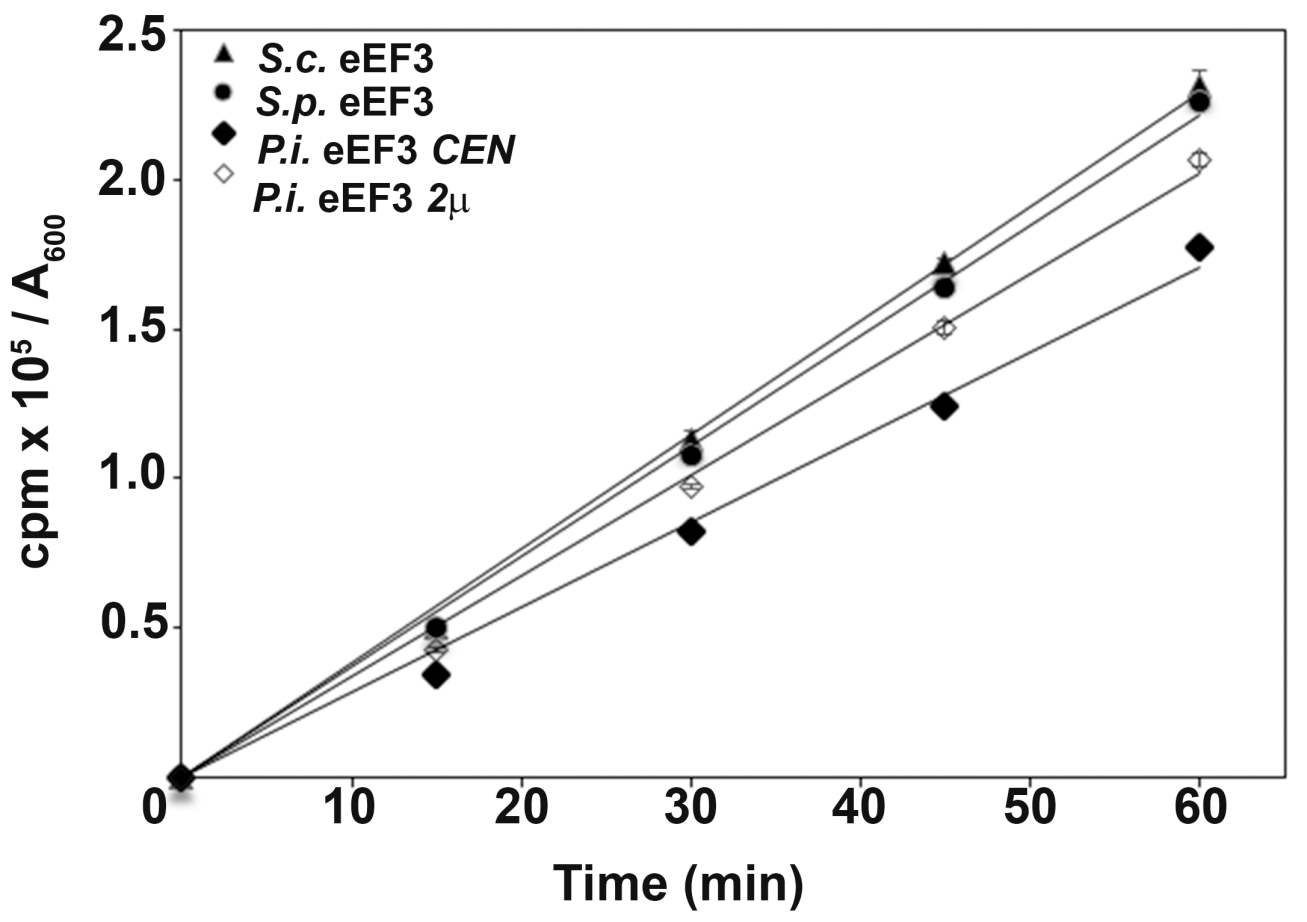
Strains expressing *P*. *infestans* eEF3 exhibit a reduction in protein synthesis. *S*. *cerevisiae* strains expressing eEF3 from the indicated species (*S*. *cerevisiae (S*.*c*.*)*, *S*. *pombe (S*.*p*.*)*, *and P*. *infestans (P*.*i*.*)* were grown to log phase in C-MET at 30°C. [^35^S] methionine was added and total protein synthesis was measured by tricholoroacetic acid precipitation. Incorporation (counts per min) is expressed per A_600_ unit. Each time point was performed in triplicate and error bars represent standard error. A representative experiment is shown.

The slow growth of *P*. *infestans* eEF3 cells compared to *S*. *pombe* expressing cells suggests that the function of *P*. *infestans* eEF3 is also compromised in the heterologous system. To determine whether the slow growth was due to a defect in translation, total protein synthesis was measured by [^35^S] methionine incorporation in exponentially growing cells expressing the indicated forms of eEF3. Strains expressing either *S*. *cerevisiae* or *S*. *pombe* eEF3 had similar rates of protein synthesis over the course of the experiment while a strain with low *P*. *infestans* eEF3 expression showed a 23–31% reduction in total protein synthesis (Fig 3). This defect was rescued to near wild-type (*S*. *cerevisiae* and *S*. *pombe)* levels by increasing the amount of *P*. *infestans* eEF3 protein with a high copy expression vector.

**Fig 4:**
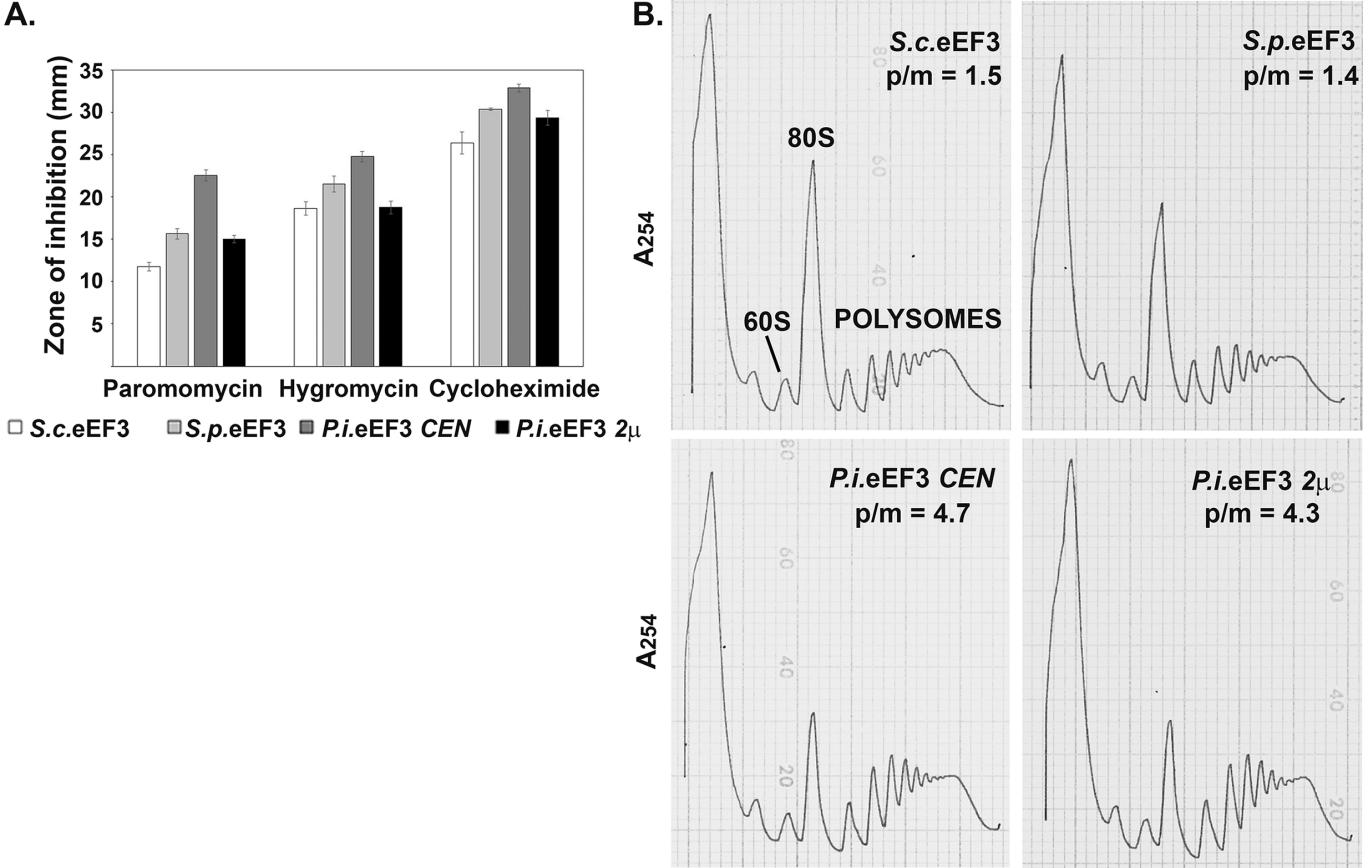
Strains expressing *P*. *infestans* eEF3 show defects in translation elongation and/or termination. (A) Antibiotic sensitivity was determined by measuring the diameter of the zone of growth inhibition around a disk containing 10 μL of the indicated drug: Paromomycin (800 mg/ml), hygromycin (25 mM), and cycloheximide (1 mM). The graph represents the average of three experiments and error bars representing the standard error are shown. (B) Ribosome extracts were prepared from the indicated strains in the absence of cycloheximide. Extracts were analyzed by 7–47% sucrose density gradient centrifugation and representative A_254_ traces are shown. The area under the 80S and polyribosome peaks was analyzed using Image J and the ratio of polyribosomes to monosomes (p/m) is indicated for each strain. *S*. *cerevisiae (S*.*c*.*)*, *S*. *pombe (S*.*p*.*)*, *and P*. *infestans (P*.*i*.*)*.

To further probe the nature of the translation defect in the low *P*. *infestans* eEF3 expression strain, sensitivity to three antibiotics known to inhibit translation elongation was measured. The low *P*. *infestans* eEF3 expression strain showed an increase in sensitivity to paromomycin, hygromycin and cycloheximide compared to wild-type cells in an assay measuring the zone of growth inhibition (Fig 4A). Increased expression of *P*. *infestans* eEF3 could either fully (hygromycin) or partially rescue (cycloheximide and paromomycin) the level of sensitivity. In these assays, *S*. *pombe* eEF3 also demonstrated a small increase in sensitivity to all three elongation inhibitors suggesting that its reduced expression level compared to *S*. *cerevisiae* eEF3 does result in a mild translation defect that does not significantly impact growth rates or total protein synthesis. To examine more specifically which steps of protein synthesis were affected in the low *P*. *infestans* eEF3 strain, polyribosome profiles were analyzed for each of the indicated strains. The polyribosome profiles of both *S*. *cerevisiae* eEF3 and *S*. *pombe* eEF3 expressing strains were similar in the absence of cycloheximide while the low *P*. *infestans* eEF3 expressing strain showed an increase in the number of polyribosomes and a concomitant decrease in the 80S ribosome peak (Fig 4B). The polyribosome profile observed in a strain expressing high levels of *P*. *infestans* eEF3, however, did not significantly differ from that seen in a strain expressing low levels of *P*. *infestans* eEF3. These results are consistent with a defect in translation elongation/termination in *P*. *infestans* eEF3 expressing strains.

### Bioinformatic analysis shows divergence in the chromodomain of *P*.*infestans* eEF3

**Fig 5:**
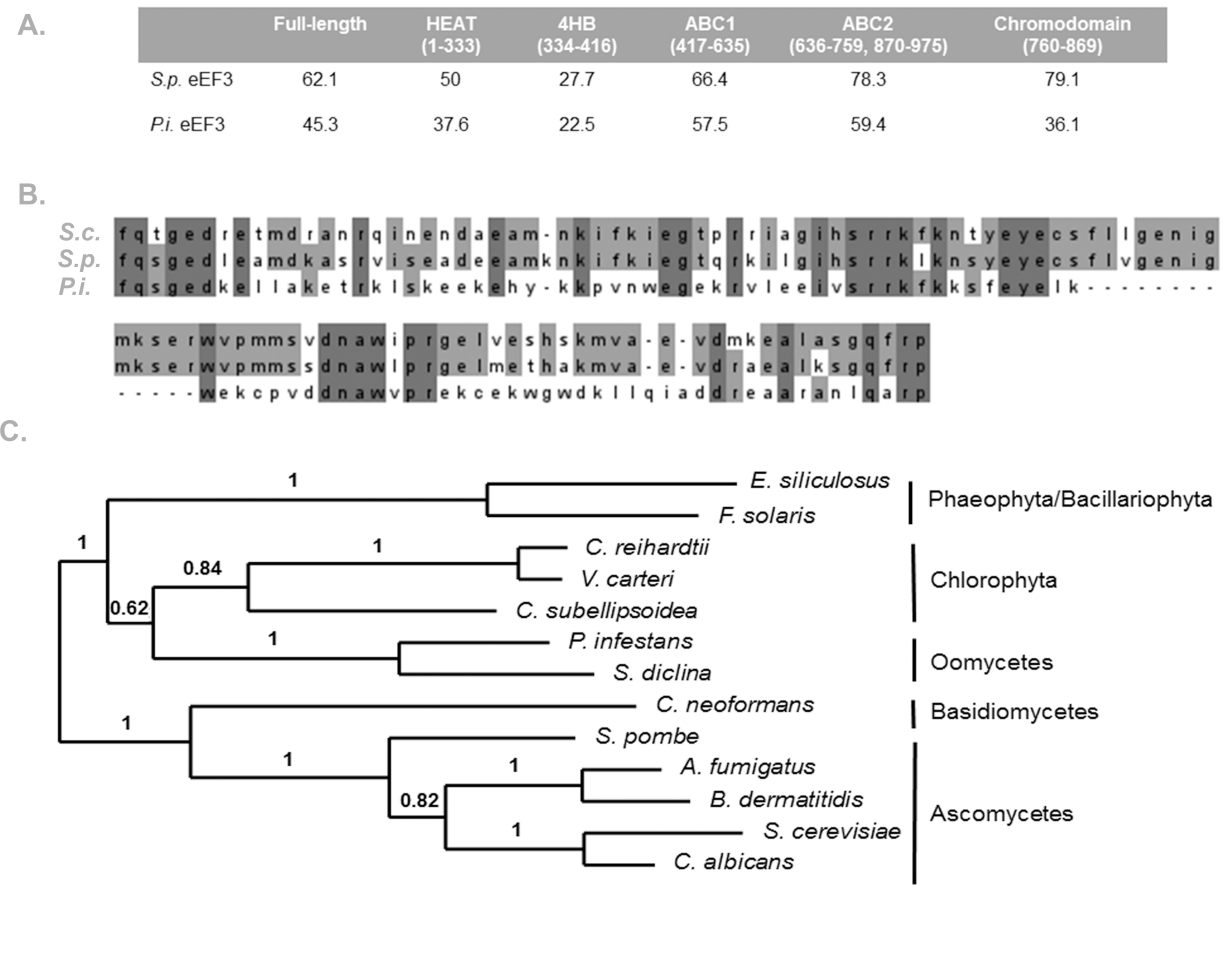
Domain specific differences in the conservation of eEF3. (A) Individual domains of *S*.*cerevisiae (S*.*c*.*)*, *S*. *pombe (S*.*p*.*)*, and *P*. *infestans (P*.*i*.*)* eEF3 were aligned using Clustal Omega and the percentage identity to *S*. *cerevisiae* eEF3 is shown [23, 24]. The amino acids comprising each domain in *S*. *cerevisiae* are indicated. (B) Alignment of the chromodomain of *S*. *cerevisiae*, *S*. *pombe*, and *P*. *infestans* eEF3 was performed using Clustal Omega and shading based on identity was done using Jalview [25]. Dark gray represents identity in all three species and light gray represents identity in two species. (C) Maximum likelihood tree with 500 bootstrap replicates created using phylogeny.fr.

To help identify the regions of eEF3 important for its activity, a domain-specific comparison of sequence identity was performed for all three eEF3 proteins (Fig 5A). As expected, the ABC1 and ABC2 domains involved in ATP binding have a high level of identity in both *S*. *pombe* eEF3 and *P*. *infestans* eEF3 when compared to *S*. *cerevisiae* eEF3. The four-helix bundle domain, whose function is currently unknown, has the lowest level of conservation across all three proteins. Outside of this domain, *P*. *infestans* eEF3 shows significantly less sequence conservation in the HEAT repeat domain and the chromodomain region, both of which have been shown to contact the ribosome in cryo-EM reconstructions [13]. The largest difference between *S*. *pombe* and *P*. *infestans* eEF3 in comparison to *S*. *cerevisiae* eEF3 is found in the chromodomain region. *S*. *pombe* eEF3 is 79.1% identical to *S*. *cerevisiae* eEF3 in this region while *P*. *infestans* eEF3 is only 36.1% identical. This region disrupts the second ABC domain and has been shown to be important for ribosome-dependent ATPase activity [10]. A sequence alignment of the chromodomain of all three eEF3s is shown in Fig 5B. Notably, *P*. *infestans* eEF3 is lacking 13-amino acids compared to both *S*. *cerevisiae* eEF3 and *S*. *pombe* eEF3. These data suggest that the chromodomain may be a species-specific determinant of ATPase activity either independently or through its role in ribosome binding.

## Discussion

The reason fungal ribosomes require eEF3 and how mammalian ribosomes have evolved to synthesize proteins without it have been long-standing questions in the field of protein synthesis. In this report, we present the first evidence that eEF3 activity exists outside the fungal kingdom. *P*. *infestans* is a filamentous eukaryotic microorganism of the oomycetes class. Oomycetes were originally grouped with fungi based on their phenotypic characteristics and are still often referred to as fungi despite genetic evidence to the contrary. Sequencing efforts have in fact shown that oomycetes are phylogenetically distinct from fungi and are instead classified with brown algae and diatoms [26, 27]. Phylogenetic comparison of another translation elongation factor, eEF1A, from *P*. *infestans* with other taxonomic groups supports the hypothesis that oomycetes evolved independently of fungi [28] and in a phylogenetic tree constructed using eEF3 sequences from a variety of unicellular eukaryotes, *P*. *infestans* eEF3 is located in the same clade as green algae and brown algae/diatoms, separate from fungi (Fig 5C). Therefore, *P*. *infestans* eEF3 is a non-fungal eEF3 which can perform the essential function of the yeast protein *in vivo*.

Despite its ability to complement the loss of *S*. *cerevisiae* eEF3, *P*. *infestans* eEF3 exhibits lower activity in the *S*. *cerevisiae* system. *S*. *pombe* eEF3 and *P*. *infestans* eEF3 are expressed at a similar level, both lower than the *S*. *cerevisiae* eEF3, but only the expression of *P*. *infestans* eEF3 is associated with an effect on growth rate. Furthermore, increasing the expression of *P*. *infestans* eEF3 using a high copy expression plasmid rescues this effect on growth. These observations correlate with what was observed *in vitro*. *P*. *infestans* eEF3 had a lower ATPase activity in the absence and presence of ribosomes. However, *P*. *infestans* eEF3 ATPase activity displayed a similar fold stimulation of activity upon the addition of ribosomes as *S*. *cerevisiae* eEF3. One explanation for these results is that it may not be an inability to interact with *S*. *cerevisiae* ribosomes but an overall low ATPase activity which causes the growth defect observed *in vivo*. Similar effects on growth rate were observed with *C*. *neoformans* eEF3 complementation. However, in this study, purified *C*. *neoformans* eEF3 activity showed weak stimulation by addition of *S*. *cerevisiae* ribosomes and an altered eEF3-ribosome interaction was suspected as the cause of the low activity [11].

The slow growth phenotype observed in *P*. *infestans* eEF3 is likely due to defects in protein synthesis. Cells with low *P*. *infestans* eEF3 expression showed a defect in [^35^S] methionine incorporation that was rescued to near wild-type levels by increasing its levels. However, increased expression of *P*. *infestans* eEF3 did not complement the polyribosome profile defect seen in cells expressing this form of eEF3. Previous experiments have shown that altered translation elongation can exist even when total protein synthesis is apparently normal [29] and may be due to the different aspects of translation being examined in each assay. These results suggest that there is still a minor elongation defect when *P*. *infestans* eEF3 is overexpressed which is consistent with the mild antibiotic sensitivities observed in this strain.

Bioinformatic analysis of existing genome databases has identified eEF3 orthologues in at least 18 different non-fungal species, including multiple green algae [14]. Since molecular genetic techniques have not been developed for many of these organisms, complementation of the loss of *S*. *cerevisiae* eEF3 may be the most expedient way to assay eEF3-like functions for these proteins, however, this technique has its limitations. For example, as noted above, heterologous eEF3 proteins are often expressed at lower levels than endogenous *S*. *cerevisiae* eEF3 from the same expression construct even when codon-optimized. We have performed similar complementation experiments using a codon-optimized construct to express *Chlamydomonas reinhardtii* eEF3 and were unable to make any conclusion on functionality because expression levels were very low even when expressed from a high copy plasmid (unpublished observations). In *S*. *cerevisiae*, it has been observed that point mutations in key Walker ATP binding motifs and the chromodomain affect the expression of eEF3 resulting in little or no protein (unpublished observations, [10]). While an effect on RNA stability cannot be excluded, these data suggest that eEF3 expression levels may be regulated by either proper folding or protein activity. In addition, lack of complementation in this system does not preclude the possibility that the heterologous eEF3 proteins retain eEF3-like function in its native organism as negative results may result from ribosomal or other divergence within the protein synthesis machinery.

The identification of eEF3 in non-fungal species will facilitate the utilization of phylogenetics to gain a greater mechanistic understanding of the role of eEF3 in translation and will provide important information on the evolution of protein synthesis. For example, the analysis of the conservation of domains among the three eEF3 proteins analyzed here, indicates that the chromodomain insertion in ABC2 maybe an important species-specific functional domain. Previous studies suggesting that this domain is important for ATPase activity [10] and the observations detailed here point to possible unique aspects of ribosome association and ATPase activation of eEF3 proteins.

Since its initial discovery as an essential, fungal-specific translation elongation factor, eEF3 has been put forth as a potential therapeutic target for the treatment of fungal infections. This work identifies a factor with eEF3 activity in another important class of pathogens. Oomycetes represent a significant threat to global food security through effects on both agriculture and fish farming [30]. If eEF3 is shown to play an essential role in oomycetes, as it does in yeast, then targeting eEF3 activity in these organisms may prove to be an effective mechanism to control these pathogens.
